# Alcohol use and intimate partner violence among women and their partners in sub-Saharan Africa

**DOI:** 10.1017/gmh.2017.9

**Published:** 2017-07-18

**Authors:** M. C. Greene, J. C. Kane, W. A. Tol

**Affiliations:** 1 Department of Mental Health, Johns Hopkins Bloomberg School of Public Health, Baltimore, MD, USA; 2 Peter C. Alderman Foundation, Kampala, Uganda

**Keywords:** Alcohol, etiology, interpersonal violence, intimate partner violence, sub-Saharan Africa

## Abstract

**Background:**

Alcohol use is a well-documented risk factor for intimate partner violence (IPV); however, the majority of research comes from high-income countries.

**Methods:**

Using nationally representative data from 86 024 women that participated in the Demographic and Health Surveys, we evaluated the relationship between male partner alcohol use and experiencing IPV in 14 countries in sub-Saharan Africa (SSA). Using multilevel mixed-effects models, we calculated the within-country, between-country, and contextual effects of alcohol use on IPV.

**Results:**

Prevalence of partner alcohol use and IPV ranged substantially across countries (3–62 and 11–60%, respectively). Partner alcohol use was associated with a significant increase in the odds of reporting IPV for all 14 countries included in this analysis. Furthermore, the relationship between alcohol use and IPV, although largely explained by partner alcohol use, was also attributable to overall prevalence of alcohol use in a given country. The partner alcohol use–IPV relationship was moderated by socioeconomic status (SES): among women with a partner who used alcohol those with lower SES had higher odds of experiencing IPV than women with higher SES.

**Conclusions:**

Results of this study suggest that partner alcohol use is a robust correlate of IPV in SSA; however, drinking norms may independently relate to IPV and confound the relationship between partner alcohol use and IPV. These findings motivate future research employing experimental and longitudinal designs to examine alcohol use as a modifiable risk factor of IPV and as a novel target for treatment and prevention research to reduce IPV in SSA.

## Introduction

Intimate partner violence (IPV), defined as any behavior within an intimate relationship that causes physical, psychological, or sexual harm, is one of the leading risk factors for mental disorder (e.g. depression, anxiety) (Trevillion *et al.* 2012), physical disorder (e.g. sexually transmitted infections, injury) (Durevall & Lindskog, 2015), and health-related disability among women of reproductive age worldwide (Krug *et al.* 2002; Shield & Rehm, 2015). The prevalence of IPV is generally higher in low- and middle-income countries (LMICs) relative to high-income countries (HICs), with some of the highest prevalence estimates identified in sub-Saharan Africa (SSA) (Garcia-Moreno *et al.* 2013), yet far less research attention is paid to IPV in these settings.

Alcohol use is a prevalent and well-established risk factor for IPV; however, evidence primarily comes from HICs (Foran & O'Leary, 2008; Abbey *et al.* 2014; Leonard & Quigley, 2017). Three theories were originally posited to explain the relationship between alcohol use and IPV (Foran & O'Leary, 2008). First, the *spurious effects model* suggests that the association between alcohol use and IPV may be explained by confounding through another factor (e.g. age, general male deviance) (Osgood *et al.* 1988; Leonard & Quigley, 1999). This model has received limited support in empirical investigations, which have failed to identify confounders that fully account for the relationship between alcohol use and IPV (Leonard, 2005). Second, the *indirect effects model* suggests that alcohol use is causally associated with IPV, but is fully mediated by other variables (e.g. relationship satisfaction) (McKenry *et al.* 1995). Third, the *proximal effects model* suggests that alcohol use directly increases aggression. The psychophysiological mechanism by which this is believed to occur is via impaired cognitive and behavioral functioning (e.g. behavioral disinhibition) (Steele & Josephs, 1990). The proximal and indirect effects models present two variations on a causal framework by which alcohol exerts an effect on IPV, which has received more empirical support relative to theories proposing non-causal relationships (e.g. spurious effects model) (Leonard, 2005). Further research has situated these causal frameworks within an ecological model, recognizing that context (i.e. individual-, partner-, community- and societal-level factors) may facilitate or prevent alcohol-related IPV (Graham *et al.* 2017). Similar to the original etiological research on this topic, these studies have largely been limited to HICs, thus making it difficult to generalize their findings to LMICs with different cultural and contextual realities.

An emerging body of literature from LMICs has identified partner alcohol use as a correlate of IPV (Hindin *et al.* 2008; Kishor & Bradley, 2012; Ezard, 2014; Durevall & Lindskog, 2015; Wagman *et al.* 2016); however, there are several limitations to this literature. First, many of the studies recruit non-representative samples and/or focus on specific subgroups of the population, making it difficult to extrapolate findings to the general population. Second, measurement of IPV and alcohol use varies substantially across contexts making it difficult to compare findings between studies. Lastly, it has been unclear how previous research in LMICs has tested the etiological theories used to explain the relationship between alcohol use and IPV.

To address these limitations, this study utilizes data from a representative sample of women of childbearing age across 14 countries in SSA to: (1) estimate the prevalence of male partner alcohol use, IPV perpetrated against female partners, and its subtypes using consistent measurement methods across countries; (2) characterize the relationship between male partner alcohol use as a hypothesized risk factor for IPV perpetrated against the female partner guided by prior etiological theories (e.g. spurious, indirect, and direct effect models); and (3) investigate whether this relationship is consistent across countries and across sociodemographic groups within countries.

## Method

### Participants

Data for this study were collected from 86 024 women aged 15–49 years in 14 SSA countries who participated in the Demographic and Health Surveys, Version 6 (DHS-6) between 2010 and 2014. The DHS-6 is a nationally representative population-based cross-sectional survey sponsored by several governmental agencies (e.g. US Agency for International Development) and administered by ICF International. The overall objective of the study is to collect, describe, and disseminate data on key demographic and health indicators for over 90 countries with a focus on HIV, nutrition, and reproductive health (The DHS Program, 2017). This study was restricted to LMICs in SSA and did not include other LMICs, given inter-regional differences in drinking patterns (e.g. higher prevalence of heavy episodic drinking in SSA) (World Health Organization, 2011), alcohol treatment and policy (World Health Organization, 2012), and cultural norms regarding alcohol use and gender (Obot & Room, 2005). Countries included in this study were: Burkina Faso (12.0%), Cameroon (4.7%), Comoros (2.8%), Cote d'Ivoire (5.7%), Democratic Republic of the Congo (6.5%), Gabon (4.4%), Malawi (6.5%), Mali (3.7%), Mozambique (7.1%), Nigeria (26.5%), Sierra Leone (5.3%), Tanzania (6.6%), Uganda (2.0%), and Zimbabwe (6.3%). Households were selected using a probability sample from census frames or, in cases where no census frame existed, from a complete list of villages or communities. Certain communities were excluded due to extreme instability or inaccessibility (ICF International, 2012). All eligible persons in a selected household were interviewed.

To address the proposed research objectives, the sample was limited to women that were selected to complete the Domestic Violence Module of the DHS-6. The response rate for women selected to complete the Domestic Violence Module was high (99.5%). Eligibility for the Domestic Violence Module included being female, 15–49 years of age, able to complete the survey privately, and currently or previously having been married and/or living with a male partner. Thus, the target population of this research is women of reproductive age in SSA that have ever been married or have lived with an intimate partner. Approximately 1% of women that were eligible and agreed to participate did not complete the interview because privacy was not possible.

### Measures

Information on basic demographics was collected from the general women's questionnaire and includes data on current marital status, urbanicity, wealth, education, literacy, and family composition. For the purpose of this analysis, marital status was classified as women who were currently married or living with a partner *v.* those who were widowed, separated, or divorced. Urbanicity was classified as urban *v.* rural. The wealth index was categorized into quintiles and was a composite variable created using principal components analysis incorporating assets, housing construction and materials, water access, and sanitation facilities (The DHS Program, 2016). Education was classified as having completed a primary education *v.* no or incomplete primary education. Lastly, each respondent was asked to read a sentence in their native language and assigned a literacy score by the interviewer. This analysis compared individuals who were completely illiterate to those with any literacy proficiency.

Data on male partner alcohol use and IPV perpetrated by the husband/male partner against the female participant were collected in the Domestic Violence Module. All questions were asked of the female participant in regards to the behaviors of her current or most recent husband/male partner. Items assessing IPV measured lifetime history of physical violence, sexual violence, and psychological violence perpetrated specifically by her current or most recent male partner. Physical violence was subdivided into less severe *v.* severe physical violence. Less severe physical violence included being slapped, pushed, shaken, having something thrown at her, having her hair pulled, or having her arm twisted. Severe forms of physical violence included being kicked, dragged, beat up, choked, burned, or threatened/attacked with a weapon. These items were based off of a modified version of the Conflict Tactics Scales (CTS) (Straus, 1990), which have been used to measure IPV in several studies in SSA (Babalola *et al.* 2014; Tlapek, 2014). The modified version of the CTS used in the Domestic Violence Module was developed to address criticisms of the CTS by including questions about sexual violence, not assuming that IPV only occurs during periods of spousal conflict, and not weighing results such that physical injury counted as more severe than other forms of IPV (Kishor & Johnson, 2006). The modified version of the CTS has shown good internal consistency for all subscales (psychological *α* = 0.79, physical *α* = 0.86, sexual *α* = 0.87) and good construct validity in HICs (Straus *et al.* 1996). To assess partner alcohol use, each participant was asked to report whether her current or most recent male partner consumed alcohol (yes/no). All measures were translated and adapted for each country then piloted in clusters not selected for inclusion in the survey to assess questionnaire quality (ICF International, 2012).

### Procedures

Interviewers were female, fluent in the local language, typically had a secondary education (or equivalent), and underwent 4–8 weeks of intensive training and supervised fieldwork. Upon successful completion of training, interviewers were assigned clusters within which they were expected to contact local authorities to solicit their cooperation during data collection after which they proceeded with recruiting selected households (ICF International, 2012). First, a household interview was conducted, which included a list of all residents. Residents that met eligibility criteria for the Domestic Violence Module were contacted to arrange a time to conduct the individual interview privately (USAID, 2013).

### Data analysis

Descriptive statistics for the following key sociodemographic characteristics were calculated for the full sample and stratified by partner alcohol use: age, marital status, urbanicity, wealth, education, literacy, and country of residence. Patterns of missingness were examined and found to be minimal (<1%) for all variables included in the analysis. We then calculated the weighted prevalence of specific types of IPV and partner alcohol use. To assess the association between partner alcohol use and IPV, we estimated a multilevel generalized linear model with a main effect of partner alcohol use controlling for age, marital status, and socioeconomic status (SES). SES was calculated by adding literacy, urbanicity, and education as items to the wealth index, which represents a composite score of household assets and characteristics (Rutstein & Johnson, 2004). The composite score, which was comprised of a single principal component, was standardized to improve interpretability.

In the first model, partner alcohol use represented the total effect of alcohol use on IPV (*β*_1*i*_), subsuming both the within- and between-country effects (model 1: LogOdds (IPV_*ij*_ = 1|*X*_*ij*_) = *β*_0*i*_ + *β*_1*i*_*X*_*ij*_ + *β*_2_*X*_Age_ + *β*_3_*X*_Marital_ + *β*_4_*X*_SES_). To disentangle the within- and between-country effects of alcohol use on IPV, we generated an estimate of alcohol use prevalence among male partners at the country level, which provides an estimate of the between-country effect of partner alcohol use on IPV (*γ*_1_). In this model, partner alcohol use may be interpreted as the within-country association with IPV (*β*_1*i*_). The difference between the between- and within-country effects represents the contextual effect at the country level. Furthermore, the variance of the random effects was examined to evaluate effect heterogeneity across countries (model 2: 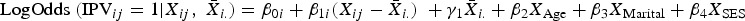). We then assessed effect measure modification by including an interaction term between SES and both alcohol exposures [partner alcohol use × SES (*β*_5_) and alcohol use prevalence × SES (*γ*_2_)] in the model (model 3: 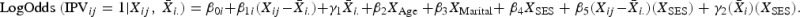 All multilevel models included a random intercept and slope to account for the correlation of observations within country. All analyses incorporated sampling weights to account for non-response and the unequal probability of selection for each participant and were estimated using the *svyset* and *gllamm* commands in Stata Version 14 (StataCorp, 2015). All graphical representations of the data were produced using R (R Core Team, 2014).

## Results

### Demographic characteristics of the sample

The 86 024 women enrolled in this study represented 80 232 women aged 15–49 years once sampling weights were applied (Table 1). On average, participants were 31.3 years of age (SD  =  8.6), most were married or living with a partner (89.7%) and living in rural regions (66.1%). Approximately 1/3 of the sample reported that their current or most recent partner uses alcohol (31.6%; 95% CI 22.2–42.8). Prevalence estimates ranged from 2.6% in Comoros (95% CI 1.8–3.8) to 62.4% in Gabon (95% CI 59.3–65.4). Participants that reported having a partner that used alcohol were older, less likely to be married, or living with their current or most recent partner, more likely to be literate and more likely to have a primary education (*p* < 0.05; Table 1).

**Table 1.** Characteristics of the sample

	All (*n* = 80 232)^a^	No alcohol use (*n* = 54 859)^a^	Alcohol use (*n* = 25 373)^a^	*t*/χ^2^	*P*
Age, M ± SD	31.3 ± 8.6	30.8 ± 8.6	32.4 ± 10.0	−5.49	<0.001
Married/living with a partner, *N* (%)	71 998 (89.7)	50 515 (92.1)	21 483 (84.7)	38.11	<0.001
Country, *N* (%)				42.0	<0.001
* Burkina Faso*	9612 (12.0)	7074 (12.9)	2537 (10.0)		
* Cameroon*	3782 (4.7)	1627 (3.0)	2155 (8.5)		
* Comoros*	2224 (2.8)	2164 (3.9)	60 (0.2)		
* Democratic Republic of the Congo*	5236 (6.5)	2567 (4.7)	2670 (10.5)		
* Gabon*	3533 (4.4)	1334 (2.4)	1299 (8.7)		
* Cote d'Ivoire*	4604 (5.7)	2991 (5.5)	1614 (6.4)		
* Malawi*	5209 (6.5)	3248 (5.9)	1961 (7.7)		
* Mali*	2954 (3.7)	2842 (5.2)	113 (0.4)		
* Mozambique*	5677 (7.1)	3463 (6.3)	2215 (8.7)		
* Nigeria*	21 230 (26.5)	17 323 (31.6)	3907 (15.4)		
* Sierra Leone*	4230 (5.3)	3529 (6.4)	702 (2.8)		
* Tanzania*	5308 (6.6)	3198 (5.8)	2110 (8.3)		
* Uganda*	1594 (2.0)	855 (1.6)	739 (2.9)		
* Zimbabwe*	5036 (6.3)	2645 (4.8)	2391 (9.4)		
Rural residence, *N* (%)	52 138 (66.1)	36 882 (68.2)	15 256 (61.5)	3.65	0.078
Literate, *N* (%)	36 848 (46.3)	22 039 (40.5)	14 809 (58.9)	16.70	0.001
Primary education, *N* (%)	31 850 (39.7)	19 121 (34.9)	12 729 (50.2)	15.91	0.002
Wealth quintile, M ± SD	3.0 ± 1.4	3.0 ± 1.4	3.1 ± 1.4	−0.60	0.560

a Weighted sample size

### Prevalence of intimate partner violence

As shown in Table 2, 42.5% of the sample experienced some form of interpersonal violence (95% CI 32.5–53.1%), the most prevalent of which was IPV. The prevalence of IPV was 36.5% (95% CI 26.7–47.7) in the sample and ranged from 10.6% in Comoros (95% CI 8.9–12.7) to 59.8% in Uganda (95% CI 56.5–62.9). Psychological violence and less severe physical violence were reported by 25.1% (95% CI 19.0–32.3) and 25.6% (95% CI 17.4–36.0) of the sample, respectively. The most common form of psychological violence was being insulted or made to feel bad by the current/most recent partner (22.4%; 95% CI 16.9–29.0). Being slapped by one's partner was the most prevalent form of less severe physical violence (22.5%; 95% CI 15.3–31.9). Although less prevalent, sexual violence (10.0%; 95% CI 6.1–16.2) and severe physical violence (8.9%; 95% CI 5.8–13.4) were reported by a notable proportion of the sample. Nine percent of the sample reported experiencing forced sex perpetrated by their partner (95% CI 5.5–14.7), which was the most common form of sexual violence. The most common form of severe physical violence was being kicked, dragged, or beat up by their partner, which was experienced by 8.0% of the full sample (95% CI 5.4–11.9).

**Table 2.** Weighted prevalence of interpersonal violence, IPV, and indicators of IPV in full sample

	Percent	95% CI
Any interpersonal violence	42.5	32.5–53.1
IPV	36.5	26.7–47.7
Non-partner family violence	11.3	8.7–14.7
Non-family violence	3.2	2.3–4.3
Any psychological IPV	25.1	19.0–32.3
Did you partner ever say or do something to humiliate you in front of others?	14.4	11.1–18.7
Did your partner ever threaten to hurt or harm you or someone you care about?	9.2	6.7–12.6
Did you partner ever insult you or make you feel bad about yourself?	22.4	16.9–29.0
Any less severe physical IPV	25.6	17.4–36.0
Did your partner ever push you, shake you, or throw something at you?	11.6	7.7–17.2
Did your partner ever slap you?	22.5	15.3–31.9
Did you partner ever twist your arm or pull your hair?	7.0	4.0–12.0
Any severe physical violence	8.9	5.8–13.4
Did your partner ever kick you, drag you or beat you up?	8.0	5.4–11.9
Did your partner ever try to choke you or burn you on purpose?	2.0	1.1–3.5
Did your partner ever threaten or attack you with a knife, gun, or other weapon?	1.5	0.9–2.4
Any Sexual IPV	10.0	6.1–16.2
Did your partner ever physically force you to have sexual intercourse with him when you did not want to?	9.1	5.5–14.7
Did your partner ever physically force you to perform any other sexual acts you did not want to?	3.7	2.0–7.0

CI, confidence interval; IPV, intimate partner violence.

### Association between alcohol use and intimate partner violence

In total, partner alcohol use (any use) was associated with a 3.2-fold increase in the odds of IPV (95% CI 2.94–3.48) controlling for age, marital status, and SES (model 1). To explore whether a contextual effect of alcohol use at the country level is present, we deconstructed the effect of alcohol use into the within- and between-country effects (Table 3; model 2). Results from this model suggest that within a given country, the odds of IPV are 122% greater for a woman who reports that her partner drinks alcohol (OR = 2.22; 95% CI 2.14–2.31). The between-country effect suggests that increasing the prevalence of alcohol use in a country by 10% is associated with a 1.4-fold increase in the average odds of IPV among women (95% CI 1.34–1.46). The contextual (country-level) effect, which is calculated as the difference in the log odds of the between- and within-country effects, suggests that for two women with the same value of partner alcohol use (i.e. yes or no), but coming from countries that differ by 10% in overall prevalence of alcohol use among partnered males, the woman from the country with the higher prevalence experiences a 59% higher odds of IPV (OR = 1.59; 95% CI 1.51–1.67). As a post hoc analysis, we evaluated whether an interaction was present between the within- and between-country effects of alcohol use, but this effect was non-significant. Examination of the random effects suggested that there was variability in the magnitude of the association between alcohol use and IPV across countries; however, the relationship between alcohol use and IPV remained consistent and robust such that 95% of countries in this region would be expected to display an association (i.e. odds ratio) between partner alcohol use and IPV between 1.43 and 3.43.

**Table 3.** Prevalence odds ratio of IPV by partner alcohol use

	Partner alcohol use (yes/no)	10% Difference in prevalence of alcohol use
	OR (95% CI)	OR (95% CI)
Model 1: total effect (*β*_1*i*_)		
	3.20 (2.94–3.48)	–
Model 2: partitioned within- (*β*_1*i*_) and between-country effect (*γ*_1_)		
	2.22 (2.14–2.31)	1.40 (1.34–1.46)
Model 3: within- and between-country effect stratified by socioeconomic status (SES)		
Main effect of alcohol (*β*_1*i*_, *γ*_1_)	2.71 (2.58–2.85)	1.35 (1.30–1.40)
Interaction with SES (*β*_5_, *γ*_2_)	0.83 (0.70–0.99)	0.98 (0.94–1.01)

Model 1: 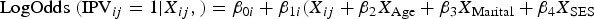.

Model 2: 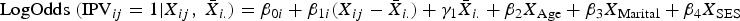.

Model 3: 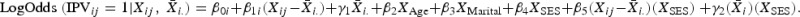

*Where partner alcohol use* = exp(*β*_1_) and prevalence of alcohol use = exp(*γ*_1_).

CI, confidence interval; IPV, intimate partner violence; SES, socioeconomic status.

**Fig. 1.**
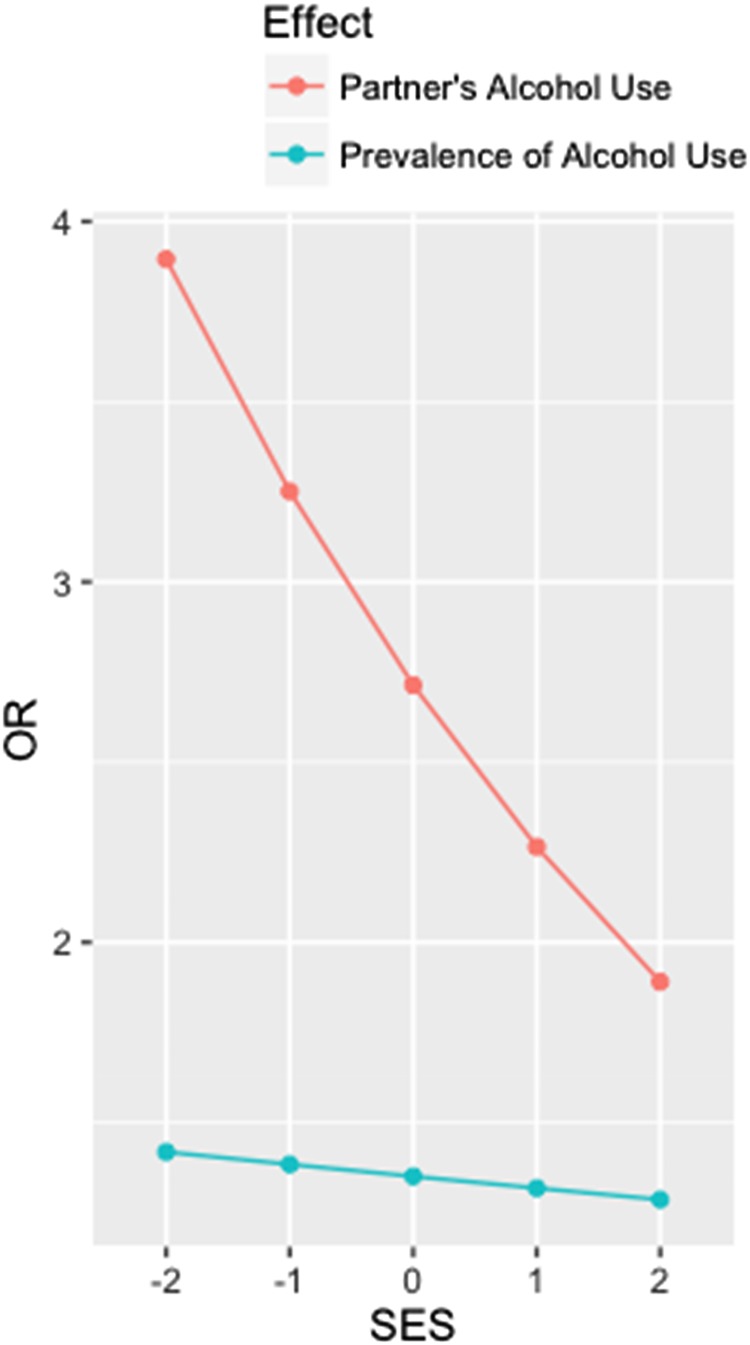
Alcohol use is associated with intimate partner violence (IPV) and the within-country effect declines with increasing socioeconomic status (SES; standardized *Z*-score). Models adjusted for age and marital status.

Upon addition of SES (standardized) as an interaction term, we found that for each standard deviation unit increase in SES, the increased odds of IPV associated with partner alcohol use was attenuated by 17% (*p*  =  0.041; Fig. 1). SES did not significantly modify the association between the prevalence of alcohol use in a country and odds of IPV. With regards to specific IPV subtypes, the increased odds of psychological, physical (less severe, severe), and sexual IPV associated with partner alcohol use was smallest for women of high SES; however, the interaction term did not achieve statistical significance (*p* > 0.05). The increase in prevalence odds associated with partner alcohol use for a given country ranged from 2.29- to 4.63-fold for sexual violence (95% CI 2.14–2.45) and less severe physical violence (95% CI 4.38–4.90), respectively (Table 4).

**Table 4.** Multivariate models estimating the prevalence odds of IPV by partner alcohol use

	Any IPV	Psychological violence	Less severe physical violence	Severe physical violence	Sexual violence
	OR (95% CI)	OR (95% CI)	OR (95% CI)	OR (95% CI)	OR (95% CI)
Partner alcohol use	2.22 (2.14–2.31)	2.38 (2.20–2.57)	4.63 (4.38–4.90)	3.43 (3.17–3.70)	2.29 (2.14–2.45)
Alcohol prevalence	1.40 (1.34–1.46)	1.18 (1.15–1.21)	1.34 (1.32–1.37)	1.32 (1.27–1.37)	1.25 (1.21–1.29)
Age	0.99 (0.99–1.00)	1.00 (0.99–1.00)	0.99 (0.99–1.00)	1.00 (0.99–1.00)	0.98 (0.98–0.99)
Marital status	0.61 (0.53–0.71)	0.60 (0.53–0.68)	0.56 (0.49–0.64)	0.46 (0.40–0.52)	0.52 (0.46–0.59)
Socioeconomic status	1.03 (0.96–1.11)	1.02 (0.96–1.07)	1.01 (0.96–1.07)	1.03 (0.95–1.12)	0.95 (0.86–1.05)

CI, confidence interval; IPV, intimate partner violence.

## Discussion

We analyzed the relationship between partner alcohol use and IPV across large representative surveys using similar methodology in 14 countries in SSA (*N* = 86 024). We found that alcohol use and IPV are prevalent public health problems in this sample. The estimated prevalence of partner alcohol use in this sample tended to be lower than the estimated prevalence of past-year alcohol use among all males 15 years and older by country as reported by the World Health Organization (2014). This may reflect a lower prevalence of alcohol use among partnered relative to non-partnered males or, alternatively, systematic differences in methods and measurement between these surveys.

In our sample, partner alcohol use was associated with increased odds of all forms of IPV (i.e. physical, psychological, and sexual) for women of reproductive age. The relationship between partner alcohol use and IPV was explained, at least in part, by both individual and contextual (country-level) factors. First, we observed a direct relationship in all 14 countries between partner alcohol use and the odds that a woman experienced IPV. Second, the odds of IPV are independently elevated for women residing in countries with a high prevalence of alcohol use. The observed between-country effect of partner alcohol use on IPV displayed a linear, dose–response relationship with few outliers. In the two outlier countries, Mali and Sierra Leone, the prevalence of IPV was higher than other countries with similar partner alcohol use prevalence estimates. Given that the prevalence of partner alcohol use is relatively low in Mali and Sierra Leone, there may be other processes that largely account for the perpetration of IPV in these settings that are not correlated with partner alcohol use to the same degree as is observed in other countries in this sample.

### Support for the indirect and direct effect models

When considering prior literature on the theoretical mechanisms by which alcohol use and IPV are related, these results align with both the proximal and indirect effects models, but not the spurious effects model (Abbey *et al.* 2014). Both the proximal and indirect effects models hypothesize that alcohol use precipitates IPV directly or indirectly via other causal mechanisms (e.g. relationship dissatisfaction, behavioral disinhibition). The spurious effects model, which hypothesizes that the relationship between alcohol use and IPV can be better explained by their relationship to a third common factor (Osgood *et al.* 1988; Leonard & Quigley, 1999), was not supported by the data included in these analyses. The most common hypothesized confounders of the relationship between alcohol use and IPV are age and social deviance. When added to the mixed-effects models, age did not confound the relationship between alcohol use and IPV. Additionally, if social deviance were to explain the association between alcohol use and IPV, we would have expected the within-country effect of partner alcohol use to be modified by the country-level overall prevalence of alcohol use, such that countries with a lower prevalence would have a stronger association between partner alcohol use and IPV than countries with higher prevalence. In our opinion, this finding would have supported the spurious effects model because alcohol use is more likely to be perceived as a deviant behavior in contexts where the prevalence is lower and it is less normative (Blum & Blum, 1969; Room, 1989; Podana & Burianek, 2013). However, we found the opposite, suggesting that prevalence of alcohol use at the country level is independent of the robust relationship between partner alcohol use and all forms of IPV. It could be hypothesized that another common factor that was not measured in this study may explain the relationship between alcohol use and IPV, such as inequitable gender norms, which has been shown to be a risk factor for both IPV and male alcohol use in SSA (Jewkes *et al.* 2010; Gottert *et al.* 2017).

These findings are consistent with prior literature, largely from HICs, that has found little evidence for the spurious effects model (Foran & O'Leary, 2008). Furthermore, empirical testing of the indirect effects model has identified partial mediators of the relationship between alcohol use and IPV, such as marital dissatisfaction, yet a direct effect typically remains. The proximal effects model has the strongest evidence base emerging from both the experimental and observational literature suggesting that the psychophysiological effects of alcohol (e.g. behavioral disinhibition) largely explain the mechanism by which individual-level alcohol use relates to IPV (Foran & O'Leary, 2008; Crane *et al.* 2016).

## Explaining the contextual effect of alcohol use and the role of socioeconomic status

An unexpected finding was the relationship between country-level prevalence of alcohol use among ever-partnered males and individual-level risk of IPV, controlling for the partner alcohol use. This suggests that drinking prevalence, which varied markedly between countries, may have an impact on IPV that is independent of individual partner alcohol use. It is possible that the contextual effect reflects relaxed social norms and policies related to both alcohol use/availability and violence (Cunradi, 2010; Cunradi *et al.* 2011), variable drinking culture and patterns between countries (World Health Organization, 2011), social disorganization and lack of community collective efficacy (Leslie *et al.* 2015), and/or the density of drinking outlets, which facilitate the assembly of high-risk groups that may socially reinforce aggressive behaviors (Gruenewald, 2007).

Overall, this finding supports the application of ecological models to describe the relationship between alcohol use and IPV as well as previous research that has identified an association between community-level social factors (e.g. neighborhood SES, community violence, and norms) and interpersonal violence (O'Campo *et al.* 1995; Raghavan *et al.* 2006; McKinney *et al.* 2009; Raghavan *et al.* 2009; Jain *et al.* 2010; Robinson *et al.* 2011; McKinney *et al.* 2012; Chong *et al.* 2015; Copp *et al.* 2015; Graham *et al.* 2017). To our knowledge, this is the first study to find an association between prevalence of alcohol use among partnered males and IPV, controlling for individual-level partner alcohol use in LMICs.

Results from the stratified analyses examining SES as an effect measure modifier suggest that the magnitude of the relationship between partner alcohol use and IPV is greater for women of low SES. Women of low SES may be more vulnerable to both the effects of partner alcohol use and IPV. However, all levels of SES display increased odds of IPV related to partner alcohol use, suggesting that alcohol use may be a risk factor for IPV across socioeconomic classes. These findings are consistent with the alcohol harm paradox (Bellis *et al.* 2016), which hypothesizes that SES is a significant moderator of the relationship between alcohol use and a variety of health outcomes (Jones *et al.* 2015).

## Limitations

The results from this study provide a foundation for exploring the relationship between alcohol use, both at the individual and country levels, and IPV in SSA. However, there are limitations of this study that should be considered when interpreting the results. First, it is possible that there are unmeasured confounders, such as cultural norms related to gender roles, which were not included in this analysis. Similarly, female partner alcohol use was not measured in the DHS-6. It is possible that IPV survivors that drink alcohol may under-report their IPV experiences due to feelings of self-blame, which could confound the relationship between male partner alcohol use and IPV (Graham *et al.* 2017).

Second, all measures, including measurement of male partner alcohol use, were reported by the female participant and thus susceptible to reporting biases. Despite this limitation, women's report of partner alcohol or drug use has been found to be a valid measurement method in previous literature on IPV using the CTS, which is the scale from which the measure of IPV and partner alcohol use in this study was derived (Lindquist *et al.* 1997). The measure of partner alcohol use was based on a single binary indicator of alcohol use *v.* no alcohol use, which is unlikely to reflect the variability in partner alcohol consumption within and across countries. Future research should improve upon these methods by applying locally validated measures of alcohol misuse to examine the relationship between partner alcohol misuse and IPV. Despite this limitation, the finding that *any* alcohol use may be associated with IPV is an important finding for intervention development. Another challenge for measurement is the cross-cultural validity of the IPV and alcohol use measures. It is possible that there are systematic differences in reporting by country. Future research should explore the cross-cultural validity of IPV and alcohol use measurement instruments in these settings.

Third, the study sample, ever-partnered women aged 15–49, represent women of reproductive age at highest risk of IPV (Garcia-Moreno *et al.* 2013); however, the results of this study should not be generalized to women outside of this age range because we are unable to infer whether our findings remain consistent in younger adolescents and older adults. Furthermore, violence reported by the ever-partnered adolescents included in this sample may also be classified as child maltreatment, which should not be considered as mutually exclusive from IPV given that girls may be partnered (formally or informally) at this age in some settings (Garcia-Moreno *et al.* 2013).

Fourth, the cross-sectional nature of this research limits our ability to make causal inferences and determine temporality in the relationship between alcohol use and IPV. We did find results that were concordant with previous literature and theoretical models supporting a causal relationship between alcohol use and IPV, but the results from this study specifically should not be interpreted as causal. Future research should apply longitudinal datasets and causal inference techniques to more rigorously test these theoretical models of the relationship, including potential mediators, between alcohol use and IPV in women and their partners in SSA. This analysis focuses specifically on IPV perpetrated by a male partner toward a female partner, which may not be generalized to all forms of IPV. Additionally, the interview asked women to report on the behaviors of their current or most recent partner, but did not specify a time frame, which may introduce the potential for recall bias.

## Strengths

Despite these limitations, this study has several notable strengths. To our knowledge, this is the first study to disaggregate the total effect of alcohol use into within- and between-country effects and explore the role of potential effect measure modifiers in SSA. Furthermore, this study utilized nationally representative data from 14 SSA countries, all of which employed the same measurement instruments allowing for improved comparisons across countries. Lastly, this is the first empirical evaluation of prevailing theories describing the relationship between partner alcohol use and IPV applied to populations in SSA.

## Conclusion

IPV affected approximately 1/3 of women of reproductive age in this sample from 14 countries in SSA, and was particularly prevalent among women whose partner drinks alcohol. There are many mechanisms that may explain this relationship as evidenced by the independent within-country, between-country, and contextual effects of partner alcohol use observed in this study. Further research employing causal inference methods and experimental designs to reduce alcohol use at the individual and community levels may build off of our findings to evaluate the causal relationship between alcohol use and IPV, identify targets for prevention and treatment interventions, and potentially reduce the burden of IPV in SSA. In addition to interventions, this research may assist in screening and identification of women affected by IPV, more specifically by recognizing that women of low SES whose partner drinks alcohol and those residing in regions with a high prevalence of alcohol use among men experience an elevated probability of experiencing IPV.

## References

[ref1] Abbey A, Wegner R, Woerner J, Pegram SE, Pierce J (2014). Review of survey and experimental research that examines the relationship between alcohol consumption and men's sexual aggression perpetration. Trauma Violence Abuse 15, 265–282. 24776459 10.1177/1524838014521031 PMC4477196

[ref2] Babalola S, Gill-Bailey A, Dodo M (2014). Prevalence and correlates of experience of physical and sexual intimate partner violence among men and women in eastern DRC. Universal Journal of Public Health 2, 25–33.

[ref3] Bellis MA, Hughes K, Nicholls J, Sheron N, Gilmore I, Jones L (2016). The alcohol harm paradox: using a national survey to explore how alcohol may disproportionately impact health in deprived individuals. BMC Public Health 16, 111. 26888538 10.1186/s12889-016-2766-x PMC4758164

[ref4] Blum RH, Blum EM (1969). A cultural case study In Drugs I: Society and Drugs (ed. R. H. Blum), pp. 226–227. Jossey-Bass: San Francisco.

[ref5] Chong VE, Lee WS, Victorino GP (2015). Neighborhood socioeconomic status is associated with violent reinjury. Journal of Surgical Research 199, 177–182. 25986212 10.1016/j.jss.2015.03.086

[ref6] Copp JE, Kuhl DC, Giordano PC, Longmore MA, Manning WD (2015). Intimate partner violence in neighborhood context: the roles of structural disadvantage, subjective disorder, and emotional distress. Social Science Research 53, 59–72. 26188438 10.1016/j.ssresearch.2015.05.001 PMC4509556

[ref7] Crane CA, Godleski SA, Przybyla SM, Schlauch RC, Testa M (2016). The proximal effects of acute alcohol consumption on male-to-female aggression: a meta-analytic review of the experimental literature. Trauma Violence Abuse 17, 520–531. 26009568 10.1177/1524838015584374 PMC4798910

[ref8] Cunradi CB (2010). Neighborhoods, alcohol outlets and intimate partner violence: addressing research gaps in explanatory mechanisms. International Journal of Environment Research and Public Health 7, 799–813. 10.3390/ijerph7030799 PMC2872327 20617004

[ref9] Cunradi CB, Mair C, Ponicki W, Remer L (2011). Alcohol outlets, neighborhood characteristics, and intimate partner violence: ecological analysis of a California city. Journal of Urban Health 88, 191–200. 21347557 10.1007/s11524-011-9549-6 PMC3079039

[ref10] Durevall D, Lindskog A (2015). Intimate partner violence and HIV in ten sub-Saharan African countries: what do the Demographic and Health Surveys tell us? The Lancet Global Health 3, e34–e43. 25539967 10.1016/S2214-109X(14)70343-2

[ref11] Ezard N (2014). It's not just the alcohol: gender, alcohol use, and intimate partner violence in Mae La refugee camp, Thailand, 2009. Substance Use and Misuse 49, 684–693. 24377756 10.3109/10826084.2013.863343

[ref12] Foran HM, O'LEARY KD (2008). Alcohol and intimate partner violence: a meta-analytic review. Clinical Psychological Review 28, 1222–1234. 10.1016/j.cpr.2008.05.001 18550239

[ref13] Gottert A, Barrington C, Mcnaughton-Reyes HL, Maman S, Macphail C, Lippman SA, Kahn K, Twine R, Pettifor A (2017). Gender norms, gender role conflict/stress and HIV risk behaviors among men in Mpumalanga, South Africa. AIDS and Behavior Vol. [epub ahead of print], 1–12. 10.1007/s10461-017-1706-9 PMC6440537 28161801

[ref56] Garcia-Moreno C, Pallitto C, Devries K, Stockl H, Watts C, Abrahams N (2013). *Global and regional estimates of violence against women: prevalence and health effects of intimate partner violence and non-partner sexual violence*, pp. 16–20. World Health Organization: Geneva.

[ref14] Graham K, Wilson I, Taft A (2017). The broader context of preventing alcohol-related intimate partner violence. Drug and Alcohol Review 36, 10–12. 27349205 10.1111/dar.12422

[ref15] Gruenewald PJ (2007). The spatial ecology of alcohol problems: niche theory and assortative drinking. Addiction 102, 870–878. 17523980 10.1111/j.1360-0443.2007.01856.x

[ref16] Hindin MJ, Kishor S, Ansara DL (2008). Intimate Partner Violence among Couples in 10 DHS Countries: Predictors and Health Outcomes. *DHS Analytical Studies No.* 18. Macro International Inc: Calverton, MD.

[ref17] ICF International (2012). Demographic and Health Survey Sampling and Household Listing Manual. Measure DHS. ICF International: Calverton, MD.

[ref18] Jain S, Buka SL, Subramanian SV, Molnar BE (2010). Neighborhood predictors of dating violence victimization and perpetration in young adulthood: a multilevel study. American Journal of Public Health 100, 1737–1744. 20634470 10.2105/AJPH.2009.169730 PMC2920975

[ref19] Jewkes RK, Dunkle K, Nduna M, Shai N (2010). Intimate partner violence, relationship power inequity, and incidence of HIV infection in young women in South Africa: a cohort study. The Lancet 376, 41–48. 10.1016/S0140-6736(10)60548-X 20557928

[ref20] Jones L, Bates G, Mccoy E, Bellis MA (2015). Relationship between alcohol-attributable disease and socioeconomic status, and the role of alcohol consumption in this relationship: a systematic review and meta-analysis. BMC Public Health 15, 400. 25928558 10.1186/s12889-015-1720-7 PMC4409704

[ref21] Kishor S, Bradley SEK (2012). Women's and Men's Experience of Spousal Violence in Two African Countries: Does Gender Matter? *DHS Analytical Studies No.* 27. ICF International: Calverton, MD.

[ref22] Kishor S, Johnson K (2006). Reproductive health and domestic violence: are the poorest women uniquely disadvantaged? Demography 43, 293–307. 16889130 10.1353/dem.2006.0014

[ref23] Krug EG, Dahlberg LL, Mercy JA, Zwi AB, Lozano R (2002). In World report on violence and health (eds. E. G. Krug, L. L. Dahlberg, J. A. Mercy, A. B. Zwi and R. Lozano), pp. 87–113. World Health Organization: Geneva.

[ref24] Leonard KE (2005). Alcohol and intimate partner violence: when can we say that heavy drinking is a contributing cause of violence? Addiction 100, 422–425. 15784050 10.1111/j.1360-0443.2005.00994.x

[ref25] Leonard KE, Quigley BM (1999). Drinking and marital aggression in newlyweds: an event-based analysis of drinking and the occurrence of husband marital aggression. Journal of Studies on Alcohol 60, 537–545. 10463811 10.15288/jsa.1999.60.537

[ref26] Leonard KE, Quigley BM (2017). Thirty years of research show alcohol to be a cause of intimate partner violence: future research needs to identify who to treat and how to treat them. Drug and Alcohol Review 36, 7–9. 27305859 10.1111/dar.12434

[ref27] Leslie HH, Ahern J, Pettifor AE, Twine R, Kahn K, Gomez-Olive FX, Lippman SA (2015). Collective efficacy, alcohol outlet density, and young men's alcohol use in rural South Africa. Health Place 34, 190–198. 26071651 10.1016/j.healthplace.2015.05.014 PMC4497916

[ref28] Lindquist CU, Sass LE, Bottomley D, Katin SM, Maddox JD, Ordonez RM, Teofilo CN (1997). Should abused women's reports of partner substance abuse be accepted as valid? Journal of Family Violence, 12, 75–83.

[ref29] Mckenry PC, Julian TW, Gavazzi SM (1995). Toward a biopsychosocial model of domestic violence. Journal of Marriage and the Family 57, 307–320.

[ref30] Mckinney CM, Caetano R, Harris TR, Ebama MS (2009). Alcohol availability and intimate partner violence among US couples. Alcoholism: Clinical and Experimental Research 33, 169–176. 10.1111/j.1530-0277.2008.00825.x PMC2692953 18976345

[ref31] Mckinney CM, Chartier KG, Caetano R, Harris TR (2012). Alcohol availability and neighborhood poverty and their relationship to binge drinking and related problems among drinkers in committed relationships. Journal of Interpersonal Violence 27, 2703–2727. 22890980 10.1177/0886260512436396 PMC3434692

[ref32] Obot IS, Room R (2005). Alcohol, Gender and Drinking Problems: Perspectives from Low and Middle Income Countries. World Health Organization: Geneva.

[ref33] O'Campo P, Gielen AC, Faden RR, Xue X, Kass N, Wang MC (1995). Violence by male partners against women during the childbearing year: a contextual analysis. American Journal of Public Health 85, 1092–1097. 7625502 10.2105/ajph.85.8_pt_1.1092 PMC1615813

[ref34] Osgood DW, Johnston LD, O'Malley PM, Backman JG (1988). The generality of deviance in late adolescence and early adulthood. American Sociological Reviews 53, 81–93.

[ref35] Podana Z, Burianek J (2013). Does cultural context affect the association between self-control and problematic alcohol use among juveniles? A multilevel analysis of 25 European countries. Journal of Contemporary Criminal Justice 29, 70–87.

[ref36] Raghavan C, Mennerich A, Sexton E, James SE (2006). Community violence and its direct, indirect, and mediating effects on intimate partner violence. Violence Against Women 12, 1132–1149. 17090690 10.1177/1077801206294115

[ref37] Raghavan C, Rajah V, Gentile K, Collado L, Kavanagh AM (2009). Community violence, social support networks, ethnic group differences, and male perpetration of intimate partner violence. Journal of Interpersonal Violence 24, 1615–1632. 19258496 10.1177/0886260509331489

[ref38] R Core Team (2014). R: A Language and Environment for Statistical Computing. R Foundation for Statistical Computing: Vienna, Austria.

[ref39] Robinson WL, Paxton KC, Jonen LP (2011). Pathways to aggression and violence among African American adolescent males: the influence of normative beliefs, neighborhood, and depressive symptomatology. Journal of Prevention & Intervention in the Community 39, 132–148. 21480031 10.1080/10852352.2011.556572

[ref40] Room R (1989). Responses to alcohol-related problems in an international perspective: characterizing and explaining cultural wetness and dryness. La ricerca Italiana sulle bevande alcoliche nel confronto internazionale, September 22–23 1989 Santa Stefano Belbo, italy.

[ref41] Rutstein SO, Johnson K (2004). The DHS Wealth Index. ORC Macro: Calverton, MD.

[ref42] Shield KD, Rehm J (2015). Global risk factor rankings: the importance of age-based health loss inequities caused by alcohol and other risk factors. BMC Research Notes 8, 231. 26054859 10.1186/s13104-015-1207-8 PMC4467665

[ref43] Statacorp (2015). Stata Statistical Software: Release 14. StataCorp LP: College Station, TX.

[ref44] Steele CM, Josephs RA (1990). Alcohol myopia. Its prized and dangerous effects. The American Psychologist 45, 921–933. 2221564 10.1037//0003-066x.45.8.921

[ref45] Straus MA (1990). Measuring intrafamily conflict and violence: the conflict tactics (CT) scales. Journal of Marriage and the Family 41, 75–88.

[ref46] Straus MA, Hamby SL, Boney-Mccoy S, Sugarman DB (1996). The Revised Conflict Tactics Scales (CTS2): development and Preliminary Psychometric Data. Journal of Family Issues 17, 283–316.

[ref47] The DHS Program. (2016). *Wealth Index* [Online]. USAID DHS: Rockville, MD. Available: http://www.dhsprogram.com/topics/wealth-index/Wealth-Index-Construction.cfm [Accessed].

[ref48] The DHS Program. (2017). *DHS Overview* [Online]. Available: http://dhsprogram.com/What-We-Do/Survey-Types/DHS.cfm [Accessed March 20 2017].

[ref49] Tlapek SM (2014). Women's status and intimate partner violence in the Democratic Republic of Congo. Journal of Interpersonal Violence 30, 2526–2540. 25315479 10.1177/0886260514553118

[ref50] Trevillion K, Oram S, Feder G, Howard LM (2012). Experiences of domestic violence and mental disorders: a systematic review and meta-analysis. PLoS ONE 7, e51740. 23300562 10.1371/journal.pone.0051740 PMC3530507

[ref51] USAID (2013). Domestic Violence Module. Demographic and Health Survey Methodology. USAID: Rockville, MD.

[ref52] Wagman JA, Donta B, Ritter J, Naik DD, Nair S, Saggurti N, Raj A, Silverman JG (2016). Husband's alcohol use, intimate partner violence, and family maltreatment of low-income postpartum women in Mumbai, India. Journal of Interpersonal Violence Vol. [epub ahead of print], 1–27. 10.1177/0886260515624235 PMC6886467 26802047

[ref53] World Health Organization (2011). Global Status Report on Alcohol and Health. World Health Organization: Geneva.

[ref54] World Health Organization (2012). Resources for the Prevention and Treatment of Substance use Disorders. [Online] World Health Organization Available: http://www.who.int/gho/substance_abuse/en/ [Accessed 2017].

[ref55] World Health Organization (2014). Global status report on alcohol and health. World Health Organization: Geneva.

